# Disease evolution in reaction networks: Implications for a diagnostic problem

**DOI:** 10.1371/journal.pcbi.1007889

**Published:** 2020-06-04

**Authors:** Abolfazl Ramezanpour, Alireza Mashaghi

**Affiliations:** 1 Medical Systems Biophysics and Bioengineering, Leiden Academic Centre for Drug Research, Faculty of Science, Leiden University, Leiden, The Netherlands; 2 Physics Department, College of Sciences, Shiraz University, Shiraz, Iran; Institute for Disease Modeling, UNITED STATES

## Abstract

We study the time evolution of symptoms (signs) with some defects in the dynamics of a reaction network as a (microscopic) model for the progress of disease phenotypes. To this end, we take a large population of reaction networks and follow the stochastic dynamics of the system to see how the development of defects affects the macroscopic states of the signs probability distribution. We start from some plausible definitions for the healthy and disease states along with a dynamical model for the emergence of diseases by a reverse simulated annealing algorithm. The healthy state is defined as a state of maximum objective function, which here is the sum of mutual information between a subset of signal variables and the subset of assigned response variables. A disease phenotype is defined with two parameters controlling the rate of mutations in reactions and the rate of accepting mutations that reduce the objective function. The model can provide the time dependence of the sign probabilities given a disease phenotype. This allows us to obtain the accuracy of diagnosis as a function of time by using a probabilistic model of signs and diseases. The trade-off between the diagnosis accuracy (increasing in time) and the objective function (decreasing in time) can be used to suggest an optimal time for medical intervention. Our model would be useful in particular for a dynamical (history-based) diagnostic problem, to estimate the likelihood of a disease hypothesis given the temporal evolution of the signs.

## Author summary

Here, we use concepts from statistical physics and reaction network dynamics to introduce a measure to quantify the tradeoff between the accuracy of diagnosis and an early diagnosis. This measure is used to suggest an optimal time for medical intervention depending on the number of observed signs (medical tests). We present a stochastic model using a reverse simulated annealing algorithm for numerical simulation of disease evolution. The model can provide the time dependence of the sign probabilities given a disease phenotype. This in turn allows us to anticipate the accuracy of diagnosis as a function of time by using a probabilistic model of signs and diseases.

## Introduction

Early diagnosis considerably reduces the human and financial cost of disease treatments. However, an early diagnosis requires an accurate characterization of disease states, understanding the mechanisms of disease development (dynamics), and the way a disease influences the other ones (disease interactions) [1–3]. This, in turn, allows us to construct more accurate diagnostic models and algorithms to uncover a hidden disease pattern in the early stages of its progress [4–8]. This study aims to clarify these concepts within a chemical reaction network as a microscopic model for the time evolution of molecular concentrations (the system signs) [9].

**Fig 1:**
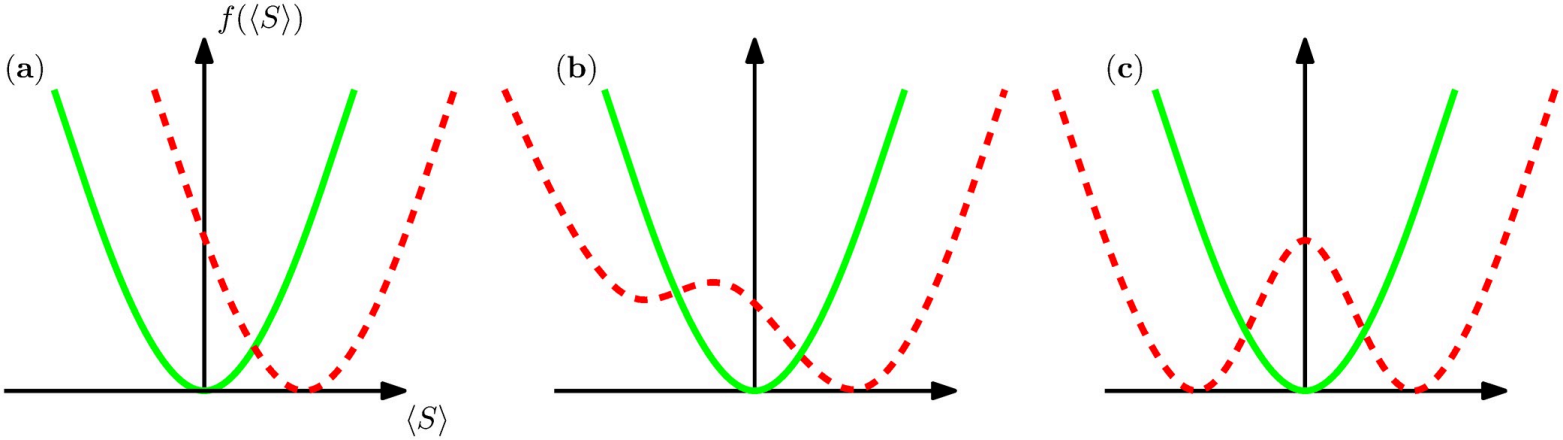
A schematic view of possible scenarios for disease development in the space of the sign probability distribution *P*(S). The average value of a binary sign variable *S*_*i*_ = ±1 is shown by 〈*S*〉. The minimums of the free energy *f*(〈*S*〉), associated with the Gibbs measure *P*(**S**), represent the possible macroscopic states of the system. (a) A healthy state changes smoothly without any phase transition. On the other hand, a disease state can emerge by a discontinuous (b) or continuous (c) phase transition. The healthy and disease cases are represented by the solid and dashed lines, respectively.

Defining a disease state and differentiating diseases based on medical signs/symptoms is not always trivial. From a statistical point of view, however, it makes sense to define the microscopic variables as the signs and define a disease state as a macroscopic (Gibbs) state of the sign probability distribution [10, 11]. A disease state then may appear by (see Fig 1): (i) changing smoothly the sign values with no phase transition, e.g., in ageing, (ii) a discontinuous phase transition, e.g., when the stress exceeds a critical value [12], (iii) a continuous phase transition which can be classified depending on its critical behaviour [13]. In recent studies, we constructed probabilistic models of signs and diseases and introduced a diagnostic algorithm that is based on the simulation of the diagnostic process [14–16]. The main finding was a two-stage diagnostic strategy, which starts by suggesting at each step one medical test and observing the outcome of that medical test (sign). Then, for a critical number of observations, the model undergoes a phase transition to an ordered phase, where it would be safe to suggest a sequence of medical tests at once, relying on the model predictions. It is very helpful here to have an accurate probabilistic model that captures the relevant statistical correlations of the sign and disease variables. A microscopic model would then be needed to construct such a probabilistic model for simulation of the diagnostic process.

The problem of disease development can be studied at different spatiotemporal resolutions. For example, the aim of molecular pathology is to understand cellular and molecular mechanisms that underlie the diseases [17]. At the level of cell population, one can study dynamics of tumor cells and their interactions with immune system with the aim of controlling the disease process [18]. Here the methods of ecological and resource-consumer theory can be used to study disease dynamics and the host-pathogen interactions [19]. At a larger scale, a complex system approach can be employed to investigate e.g. the neural dynamics of neurological disorders [20]. On the other hand, clinical data acquisition and monitoring of disease dynamics are essential for understanding of disease development [21, 22]. The methods of complex dynamic systems and machine learning can be employed here to analyse the data and construct reasonable dynamical models.

In this paper, we are going to study the time evolution of disease states in a simple model of biochemical reactions. Unfortunately, the concepts of signs, symptoms, and diseases are not always well defined with clear boundaries. This will expectedly change in the future when high resolution molecular picture of diseases are available, thanks to advances in multi-omics approaches. The use of a reaction network as a specific microscopic model provides clear definitions for the above concepts. We take the number of molecules as the microscopic or sign/symptom variables. Here the symptoms are equivalent in meaning to the signs and are used interchangeably. The value of an observed sign (as a medical test) reveals the number or concentration of a biochemical species. The reactions here play the role of interactions between the microscopic variables and the reaction rates give the strength of these interactions. On the other hand, we define the diseases or disease phenotypes as the macroscopic or emergent behaviours displayed by the sign variables. A defect in the reaction network is defined below as a specific deviation in the reaction rates of the healthy network. One may consider a defect as a simple and well defined disease, but we stress that in general a disease is defined by the collective contributions of the signs with (possibly) multiple defects.

We introduce an effective dynamics for temporal evolution of diseases, which is inspired from thermal annealing of physical systems to reach a low temperature (ordered) state of the system. We apply it to a specific model of microscopic signs (molecular concentrations in a reaction network) to study disease development and its consequences for disease diagnosis in different stages of the process. A reaction network provides us with a testbed to model disease developments and disease-disease interactions, and follow the response of the sign variables (here molecular concentrations) as the time pass [23–25]. Specifically, we start from a healthy reaction network which maximizes the mutual information between a subset of signal and response variables as the objective function of the system [26–28]. The optimization step can be done by a local optimization algorithm like the simulated annealing algorithm [29]. Then, we introduce some defects (mutations) in the system and run the simulated annealing algorithm in the reverse direction, which on average decreases the objective function of the system. The system dynamics here is controlled by the rates of introducing the defects and the rate of accepting a decrease in the objective function. Note that by maximizing the mutual information in the healthy state, the system could be placed in a critical region close to a phase transition point. And, the reverse annealing algorithm can induce a phase transition from the healthy state (say an ordered phase) to a disease state (disordered phase). Another aim of this study is to provide a microscopic model of time-dependent signs and diseases which can be used for diagnosis from the history or dynamics of the observed signs, and which in addition allows us to model disease-disease interactions more explicitly.

As a first step, in this study we use the synthetic data generated by the above dynamical model to investigate the time dependence of the diagnostic performance with a simple probabilistic model of signs and diseases. We see how the accuracy of diagnosis with such a diagnostic model improves with time as the reaction network deviates from the healthy state of maximum objective function. This information would be necessary for quantification of the tradeoff between the accuracy of diagnosis and the level of disease progression at the time of diagnosis. This, in particular, can be used to suggest an optimal intervention or screening time for specific diseases and diagnostic models.

The paper is structured as follows. In Sec The model and definitions we define the model and give the main equations. In Sec Modelling disease evolution we present the stochastic model of disease evolution, and the results of numerical simulations for a small reaction network. The concluding remarks are given in Sec Discussion.

## Results

### The model and definitions

**Fig 2:**
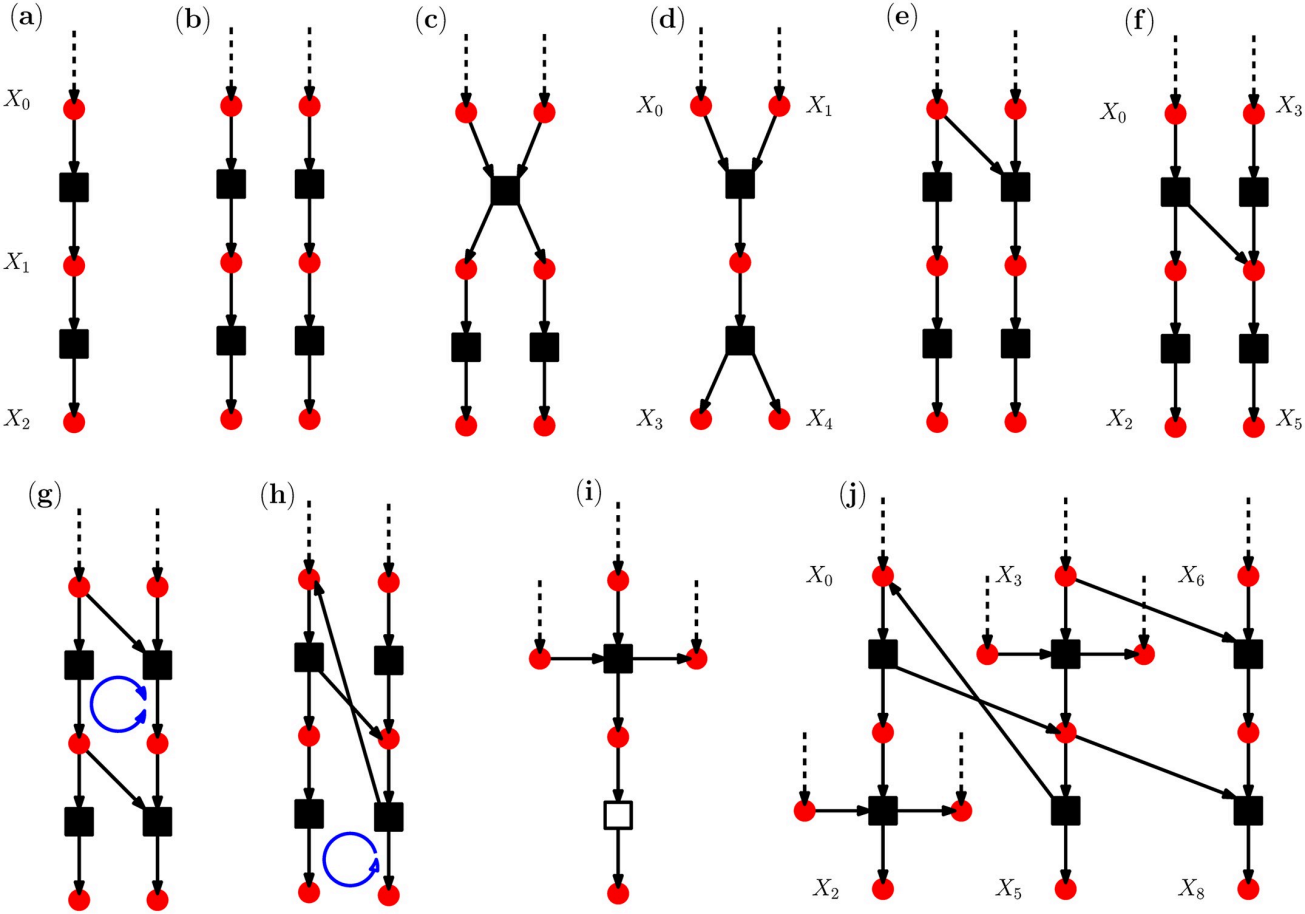
Illustration of the reaction networks. The solid circles and squares display the species and the reactions, respectively. A reaction could be reversible (full square) or irreversible (empty square). A dashed arrow shows that the species concentration is driven externally. (a) a single pathway, (b) two non-interacting pathways, (c)-(d) two pathways interacting through common reactions and species, (e)-(f) two pathways interacting through a link, (g)-(h) two pathways interacting through a coherent (h) or incoherent (g) cycle, (i) a pathway with an irreversible reaction which also interacts with two externally driven species, (j) a larger reaction network of interacting pathways with *M* = 6 reactions and *N* = 13 species.

We consider a system of *N* interacting species (molecules) with *M* chemical reactions [9, 30]. Fig 2 displays a graph representation of some elementary reaction networks. A reaction network can be represented by a number of reaction pathways as the fundamental building blocks (basis) of the reaction network [26]. We use the integers 0 ≤ *X*_*i*_(*t*) ≤ *X*_*max*_ to show the number of molecules for species *i* = 1, …, *N* at time *t*. Here *X*_*max*_ is the maximum number of molecules that is allowed by the biological system. The whole set of molecular numbers are denoted by vector **X**(*t*) = {*X*_1_(*t*), …, *X*_*N*_(*t*)}. To be specific, let us assume that our reaction network is a signal transduction network, where the signals are encoded in the temporal concentration of some signal species. The set of signal variables is denoted by S. To each signal species i∈S we assign the subset of associated response species R(i). The signal generated by species *i* is transmitted through the network to species j∈R(i). This means that a change in the concentration of signal molecules is expected to significantly affect the activation level of the response molecules.

In general, a reaction *r* = 1, …, *M* is represented by the stoichiometric coefficients $\nu_{r}^{-}\left(i\right)$ and $\nu_{r}^{+}\left(i\right)$

$$\begin{array}{c} \sum_{i=1}^{N}\nu_{r}^{-}\left(i\right)X_{i}\to \sum_{j=1}^{N}\nu_{r}^{+}\left(j\right)X_{j}. \end{array}$$ (1)

The coefficients $\nu_{r}^{-}\left(i\right),\nu_{r}^{+}\left(i\right)$ take only non-negative integer values. The reactions happen stochastically with a probability that is determined by a propensity function *η*_*r*_(**X**). From the stochastic simulation of this process one can extract the probability distribution of the number of species. Let Pr(*X*_*i*_, *X*_*j*_) be the joint probability distribution of variables *X*_*i*_ and *X*_*j*_ in a stochastic process governed by the above reactions. The mutual information of the two variables, which measures any statistical dependency of the variables, is given by

$$\begin{array}{c} \text{MI}\left(i,j\right)=\sum_{X_{i}}\sum_{X_{j}}\text{Pr}\left(X_{i},X_{j}\right)\log\frac{\text{Pr}(X_{i},X_{j})}{\text{Pr}\left(X_{i}\right)\text{Pr}\left(X_{j}\right)}. \end{array}$$ (2)

We need the above measure later to quantify the degree of statistical correlations between the signal and response variables.

The average value of a single variable is denoted by

$$\begin{array}{c} \left\langle X_{i}\right\rangle =\sum_{X_{i}}X_{i}\text{Pr}\left(X_{i}\right). \end{array}$$ (3)

The activation level of species *i* can be represented by a coarse-grained variable *x*_*i*_ ∈ {−1, 0, +1}, where

$$\begin{array}{ll} x_{i}=-1 & \text{if}\langle X_{i}\rangle <X_{i}^{*}-\delta X_{i}\\ x_{i}=+1 & \text{if}\langle X_{i}\rangle >X_{i}^{*}+\delta X_{i} \end{array}$$

otherwise *x*_*i*_ = 0. Later, we use these variables as the signs in a probabilistic model of disease diagnosis. Here $X_{i}^{*}$ defines the threshold value for variable *i* and *δX*_*i*_ denotes the standard deviation in *X*_*i*_. We shall set $X_{i}^{*}=X_{max}/2$. The threshold value here is defined according to the number of coarse-grained levels, but in practice its precise value would depend on the biological details of the system.

The linear correlation coefficient of two variables is defined as follows

$$\begin{array}{c} C\left(i,j\right)=\frac{\left\langle X_{i}X_{j}\right\rangle -\left\langle X_{i}\right\rangle \left\langle X_{j}\right\rangle }{\sqrt{\left\langle {\left(X_{i}-\left\langle X_{i}\right\rangle \right)}^{2}\right\rangle \left\langle {\left(X_{j}-\left\langle X_{j}\right\rangle \right)}^{2}\right\rangle }}. \end{array}$$ (4)

Note that in general the mutual information MI(*i*, *j*) gives more information about the statistical dependence between the variables than the above linear correlations. Nevertheless, it is easy to see from the sign of *C*(*i*, *j*) that the two variables are positively or negatively correlated. The above definitions give the basic statistical measures that are used in the following to characterize the statistical state of the system.

#### Evolution by the stochastic chemical kinetics

The system evolves in time by *M* (reversible or irreversible) chemical reactions, where *X*_*i*_(*t*) gives the number of species *i* at time *t*. Each chemical reaction *r* = 1, …, *M* is identified with the state-change vectors (stoichiometric coefficients) $\nu_{r}^{-},\nu_{r}^{+}$ and the propensity function *η*_*r*_(**X**), which determines the reaction flux and in general depends on the *X*_*i*_. The state-change vectors give the changes in the number of molecules; i.e., after reaction *r* the *X*_*i*_ change to $X_{i}+\left(\nu_{r}^{+}\left(i\right)-\nu_{r}^{-}\left(i\right)\right)$. The propensity function *η*_*r*_(**X**)*dt* gives the probability that reaction *r* happens in the time interval (*t*, *t* + *dt*) given the **X**(*t*) = {*X*_1_(*t*), …, *X*_*N*_(*t*)}. Note that each reaction happens with the above probability independently of the other reactions as in a Poisson process [31]. However, a single specie may be involved in several reactions affecting the dynamics of the associated reactions. The propensity function depends on the number of molecules in the left hand side of the reaction $L_{r}=\left\{i:\nu_{r}^{-}\left(i\right)>0\right\}$. The propensity function can take different forms depending on the nature of reaction. For instance, within the mass-action kinetics [9]:

$$\begin{array}{c} \eta_{r}\left(X\right)=\kappa_{r}\prod_{i\in L_{r}}\frac{X_{i}!}{\nu_{r}^{-}\left(i\right)!\left(X_{i}-\nu_{r}^{-}\left(i\right)\right)!}, \end{array}$$ (5)

where the reaction rate *κ*_*r*_ scales with the system volume Ω as $1/\Omega^{|L_{r}|-1}$. It is assumed that the molecules are confined in a bounded region of space denoted by Ω. The scaling of the reaction rates with the volume is to ensure that *X*_*i*_/Ω is well defined in the thermodynamic limit Ω → ∞.

The average values of stochastic variables *X*_*i*_(*t*) satisfy the following equation

$$\begin{array}{c} \frac{d\langle X_{i}\left(t\right)\rangle }{dt}=\sum_{r}\left(\nu_{r}^{+}\left(i\right)-\nu_{r}^{-}\left(i\right)\right)\left\langle \eta_{r}\left(X\right)\right\rangle . \end{array}$$ (6)

We see that even solving for the averages 〈*X*_*i*_(*t*)〉 is difficult due to the coupling of the variables in the right hand side of the equation. In the following, however, we resort to a numerical simulation of the above process. By simulating a large population of identical reaction networks we also obtain the joint probability distributions which are needed for computation of the mutual informations.

One can use the Stochastic Simulation Algorithm (or Gillespie algorithm) to simulate the time evolution of a reaction network [32]. Let us assume that a reaction happened at time *t*. Then, we need to know the time to the next reaction (*τ*) and the index of the next reaction (*r*). Given that the system is currently in state **X**(*t*), the joint probability distribution of two random variables *ρ*(*τ*, *r*) is:

$$\begin{array}{c} \rho \left(\tau ,r\right)=\eta_{r}\left(X\right)e^{-\eta (X)\tau },\;\eta \left(X\right)\equiv \sum_{r}\eta_{r}\left(X\right). \end{array}$$ (7)

Note that no reaction should happen in the time interval *τ*. That is why we need the exponential factor with the total propensity function *η*(**X**). The Gillespie algorithm then goes as follows [32]:

- compute the *η*_*r*_ and *η* given the **X**(*t*)
- extract *τ* and *r* from the probability distribution *ρ*(*τ*, *r*):
  - draw two random numbers *u*_1_, *u*_2_ from the uniform distribution in (0, 1)
  - then $\tau =\frac{1}{\eta }\log\left(1/u_{1}\right)$ and *r* is the smallest integer satisfying $\sum_{r^{\prime }=1}^{r}\eta_{r^{\prime }}>u_{2}\eta$
- change *t* → *t* + *τ* and $X\to X+(\nu_{r}^{+}-\nu_{r}^{-})$

Note that we should always check to have 0 ≤ *X*_*i*_ ≤ *X*_*max*_. That means that a reaction should have enough reactants to happen, and we keep the *X*_*i*_ ≤ *X*_*max*_.

This is a simple but very time consuming algorithm for simulation of large reaction networks. The reader can refer to other references in [32] and [33, 34] for more efficient and sophisticated algorithms.

### Modelling disease evolution

In this section, we introduce a simple dynamical model for the time evolution of defects in the reaction network. We shall assume that the number of species and the pattern of interactions by the reactions are fixed. Moreover, we assume that the healthy state is an optimal state for an appropriately defined objective function, which depends on the nature of functions we expect from the system. For instance, if the main task of a system is to remember a number of patterns, then a good objective function could be the number or quality of the stored patterns. A reaction network of interacting pathways can be considered as a signal transaction and processing system, among the other functions [26–28]. To be specific, in the following we assume that the healthy state is defined by the state of maximum mutual information between the signal variables and the associated responses. A more suitable measure is the transfer entropy from the signal to response variables. This is a directed measure of information transmission (or causal relation) which is expected from the signal transduction part of the reaction network in a cell. Mutual information is however a symmetric measure of statistical dependence which is closely related to the transfer entropy and at the same time it is computationally easier to estimate; because here one needs two-point joint probabilities whereas for computation of transfer entropy one needs three-point conditional probabilities.

Given the structure of the reaction network, we first use an approximate optimization algorithm (e.g. a zero-temperature Monte Carlo) to find the reactions rates that maximize the following objective function:

$$\begin{array}{c} E\equiv \sum_{i\in S}(\frac{1}{\left|R(i)\right|}\sum_{j\in R(i)}\text{MI}\left(i,j\right)). \end{array}$$ (8)

Note that the mutual information are obtained from the stochastic simulation of the species dynamics which depends on the reaction rates. Therefore, one can search in the space of the reaction rates to find the optimal parameters that maximize the mutual information and so the objective function.

We start from the stationary state of the optimal (healthy) state and denote the associated objective function value by $E_{old}$. We also attribute an elementary defect ${\hat{d}}_{r}$ to each reaction which means that the associated reaction rate is deviating from the healthy value, e.g. due to mutations in the related genes. A general defect pattern is obtained by a combination (or a set) of these elementary defects $D=\sum_{r=1}^{M}D_{r}{\hat{d}}_{r}$. The number of nonzero coefficients *D*_*r*_ = 0, 1 gives the number of present elementary defects in **D**, which is denoted by |**D**| (cardinality of set **D**). We call **D** a defect pattern because in general it can be a superposition of multiple elementary defects. Then, the time evolution of a defect (or pathologic variation) pattern **D** is modelled in the following way: For *t* = 1, …, *t*_*max*_ do:

- each reaction rate changes with (mutation) probability *α*_*r*_(*t*|**D**) from *κ*_*r*_ to *κ*_*r*_ ± *δκ*
- run the stochastic simulation (Gillespie) algorithm to compute the new objective function $E_{new}$
- accept the changes in the reaction rates if the objective function increases, otherwise, accept the changes with probability exp(β(t|D)ΔE). If the changes are accepted set $E_{old}=E_{new}$

The mutation probabilities *α*_*r*_(*t*|**D**) are expected to be negligible at the beginning (for a healthy state) and increase with time step *t*. This probability is greater than zero only for the elementary defects that are presented by **D**. For instance, in case that only elementary defect *D*_*a*_ is present, the mutation probability is denoted by *α*_*r*_(*t*|*D*_*a*_) which is nonzero only for *r* = *a*. On the other hand, the parameter *β*(*t*|**D**) in the acceptance probabilities is expected to be very large for a healthy state and decrease with *t* depending on **D**. Here *β*(*t*|**D**) ≥ 0 plays the role of an inverse temperature in a reverse simulated annealing algorithm [29]. While the probabilities *α*_*r*_(*t*|**D**) control the rate of local changes (mutations) in the reaction network, the global parameter *β*(*t*|**D**) determines the system susceptibility to such local mutations (as the immune system). Note that in the standard simulated annealing the temperature decreases slowly to reach an optimal state of the system, which maximizes the objective function (minus the energy function). Here, however, we are deviating from the optimal state by increasing a temperature-like parameter 1/*β*. That is why we call the above process a reverse annealing.

Let *τ*_*r*_(**D**) denote the time scale in which *α*_*r*_(*t*|**D**) changes from zero to one. Similarly we define the time scale *τ*_*β*_(**D**), for changing the probability of accepting a decrease in the objective function. The rates 1/*τ*_*r*_(**D**) could in general be nonzero for an arbitrary subset of the reactions.

Interactions between two evolving defects **D**_*a*_ and **D**_*b*_ may change the rate of mutations and acceptance probabilities, for instance, by

$$\begin{array}{c} \frac{1}{\tau_{r}\left(D_{a}+D_{b}\right)}=\frac{1}{\tau_{r}\left(D_{a}\right)}+\frac{1}{\tau_{r}\left(D_{b}\right)}+\frac{\lambda_{r}^{ab}}{\tau_{r}\left(D_{a}*D_{b}\right)}, \end{array}$$ (9)

$$\begin{array}{c} \frac{1}{\tau_{\beta }\left(D_{a}+D_{b}\right)}=\frac{1}{\tau_{\beta }\left(D_{a}\right)}+\frac{1}{\tau_{\beta }\left(D_{b}\right)}+\frac{\lambda_{\beta }^{ab}}{\tau_{\beta }\left(D_{a}*D_{b}\right)}. \end{array}$$ (10)

The additional rates 1/*τ*_*r*_(**D**_*a*_ * **D**_*b*_), 1/*τ*_*β*_(**D**_*a*_ * **D**_*b*_) and couplings $\lambda_{r}^{ab},\lambda_{\beta }^{ab}$ show how much the two defects alter the reactions that are not directly affected by the single defects. Here, we use the fact that the rate of two independent Poisson processes with rates 1/*τ*_1_ and 1/*τ*_2_ is given by 1/*τ* = 1/*τ*_1_ + 1/*τ*_2_. Then, a deviation from this case, due to an interaction between the two processes, is represented by an additional term λ_12_/*τ*_12_. Note that **D**_*a*_ * **D**_*b*_ is not a simple multiplication; it is just a notation we introduced to represent the interaction between the two defects.

#### Evaluation of diagnosis accuracy

Given a defect pattern **D**, we can compute the molecular numbers *X*_*i*_(*t*) and the associated mutual information MI(*i*, *j*) and correlations *C*(*i*, *j*) at each time step *t* of the disease evolution algorithm described above. Here, we are going to map the coarse-grained variables *x*_*i*_ to binary sign variables **S** = {*S*_*i*_ = ±1: *i* = 1, …, *N*} in a diagnostic problem. The aim here is to find out the underling defects **D**, assuming that we have observed the values of *N*_*O*_ ≤ *N* signs at time *t*. We can compute the conditional defect probabilities from a probabilistic model Pr(**D**, **S**) of the sign and defect variables given the observed signs. Then, the most probable defect pattern is taken for the diagnosis. A computationally simple model (call it the D1S1 model) is obtained by assuming that the elementary defects are marginally independent of each other Pr(**D**) = ∏_*a*_ Pr(*D*_*a*_), and the signs are conditionally independent of each other Pr(**S**|**D**) = ∏_*i*_ Pr(*S*_*i*_) [5–7].

In the following, we shall use the above model for diagnosis, assuming that the number of present defects |**D**| is one. This simplification is to avoid the unnecessary complications of the diagnosis problem and focus on the temporal evolution of diagnosis accuracy. To construct the D1S1 model (i.e. to set the model parameters), we need the conditional probabilities Pr_*t*_(*S*_*i*_|nodefect) and Pr_*t*_(*S*_*i*_|*D*_*r*_), in the healthy state (with no defect) and in presence of only one elementary defect (*D*_*r*_) [14]. This information is obtained (for all the *D*_*r*_) from the disease evolution algorithm as describe above.

To evaluate the model predictions, first we run the disease evolution algorithm for a given elementary defect *D*_*r*_. This results to the associated sign values *S*_*i*_(*t*) (obtained from the molecular numbers) for different times *t*. Then, it is assumed that we know only the value of *N*_*O*_ signs (the observed signs) which is used by the diagnostic model (D1S1). From this model we infer the most probable defect and take it as the model prediction, which can be compared with the true one *D*_*r*_. In this way, we obtain the accuracy of diagnosis by the D1S1 model, conditioned on the number of observed signs:

$$\begin{array}{c} AC\left(t:N_{O}\right)=\frac{P_{true}+N_{true}}{P_{total}+N_{total}}. \end{array}$$ (11)

This is the ratio of true positive (*P*_*true*_) and true negative (*N*_*true*_) to the total number of positive (*P*_*total*_) and negative (*N*_*total*_) cases. Obviously 0 ≤ *AC*(*t*: *N*_*O*_) ≤ 1 and *AC*(*t*: *N*_*O*_) = 1/2 for a completely random diagnosis. Moreover, the accuracy is expected to increase with the observation time at which the *N*_*O*_ signs are measured; the larger time *t* the more specific are the observed signs regarding the involved defects.

**Fig 3:**
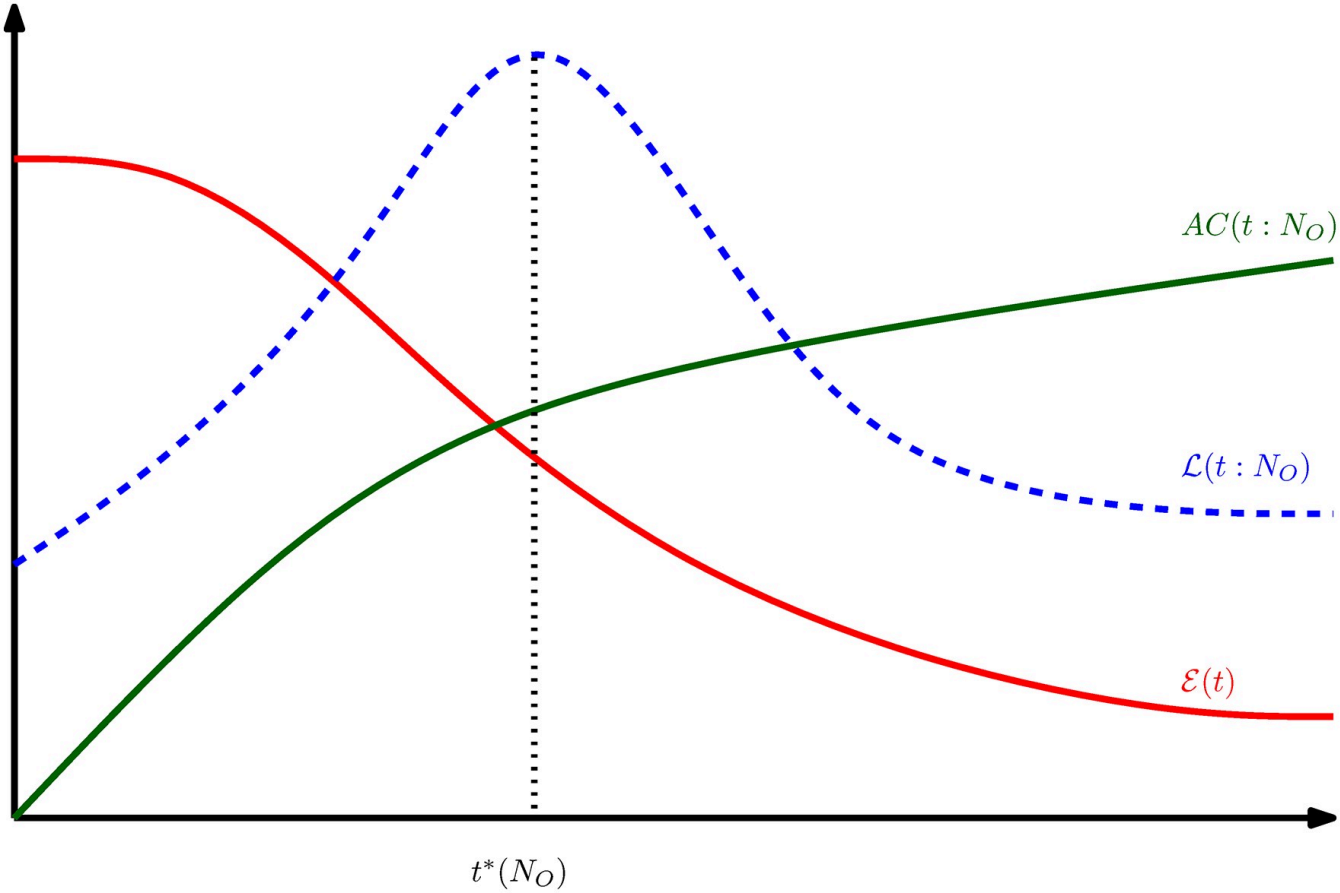
A schematic behaviour of disease progress and diagnosis accuracy with time. An optimal intervention time t*(*N*_*O*_) can be defined by maximizing another measure $L(t:N_{O})$ constructed by the objective function E(t) plus the diagnosis accuracy *AC*(*t*: *N*_*O*_), given the number of observed signs *N*_*O*_, and the number of present diseases |**D**|.

Now, imagine that we are to find the best time for observing *N*_*O*_ signs assuming that |**D**| = 1. In one hand, we have the objective function E(t) which is a decreasing function of time and shows the disease progress. On the other hand, we have the diagnosis accuracy *AC*(*t*: *N*_*O*_) which improves with the disease progress in time. An optimal intervention time t*(*N*_*O*_) then can be obtained by maximizing a weighted sum of the two functions:

$$\begin{array}{c} L\left(t:N_{O}\right)\equiv \lambda \frac{E(t)}{E(0)}+\left(1-\lambda \right)\frac{\left(AC\left(t:N_{O}\right)-\frac{1}{2}\right)}{1/2}, \end{array}$$ (12)

using the normalized quantities (divided by the maximum values) with 0 ≤ λ ≤ 1. The value of λ determines the importance that we associate with each term. In practice, the λ value is gradually adjusted in a learning process to find the optimal value, which could depend on the number of present defects |**D**| and the nature of diseases. Fig 3 displays schematically the expected behaviour of the above quantities. In practice, we do not have access to the future values of the objective function and the unobserved signs to compute the diagnosis accuracy. But, we can use the microscopic model of disease evolution (here the reaction network) in addition to the probabilistic diagnostic model (here the D1S1 model) to simulate the above process and find an estimation of the optimal intervention time. This highlights the importance of simulation-based methods in a diagnostic problem.

Note that here for simplicity we assume that the number of involving (present) elementary defects is one (|**D**| = 1). In addition, more accurate (and sophisticated) diagnostic models can be used by considering the possibility of direct defect-defect and sign-sign interactions in the probabilistic model. Such models could be very useful to deal with the case that multiple interacting defects are at work [14]. Finally, the stochastic dynamics of the reaction network and the defects in the disease evolution algorithm provides an ensemble of sign histories which are consistent with a given defect pattern **D**. This statistical information would be very useful for having a more accurate diagnosis considering the effect of such disorders on the system dynamics.

#### Numerical simulations

In this section we present the results of numerical simulation for the reaction networks of Fig 2. For example, consider the reaction network of Fig 2(j) with three interacting pathways, *N* = 13 species, and *M* = 6 reversible reactions. We assume that each reaction pathway has its own signal and response species; the selection of these variables in general depends on the specific function of the reaction network which is considered. Here, the signal variables and the associated responses are: $S=\{X_{0},X_{3},X_{6}\}$, $R\left(X_{0}\right)=X_{2},R\left(X_{3}\right)=X_{5},R\left(X_{6}\right)=X_{8}$. And, the objective function is

$$\begin{array}{c} E=\text{MI}\left(X_{0},X_{2}\right)+\text{MI}\left(X_{3},X_{5}\right)+\text{MI}\left(X_{6},X_{8}\right). \end{array}$$ (13)

We use a zero-temperature Monte Carlo algorithm to find a local maximum of the objective function by changing only the reaction rates, with the constraint that *κ*_*r*_ ∈ (0, 2); this range of values was chosen such that the models can display different non-trivial phases in a reasonable computation time.

**Fig 4:**
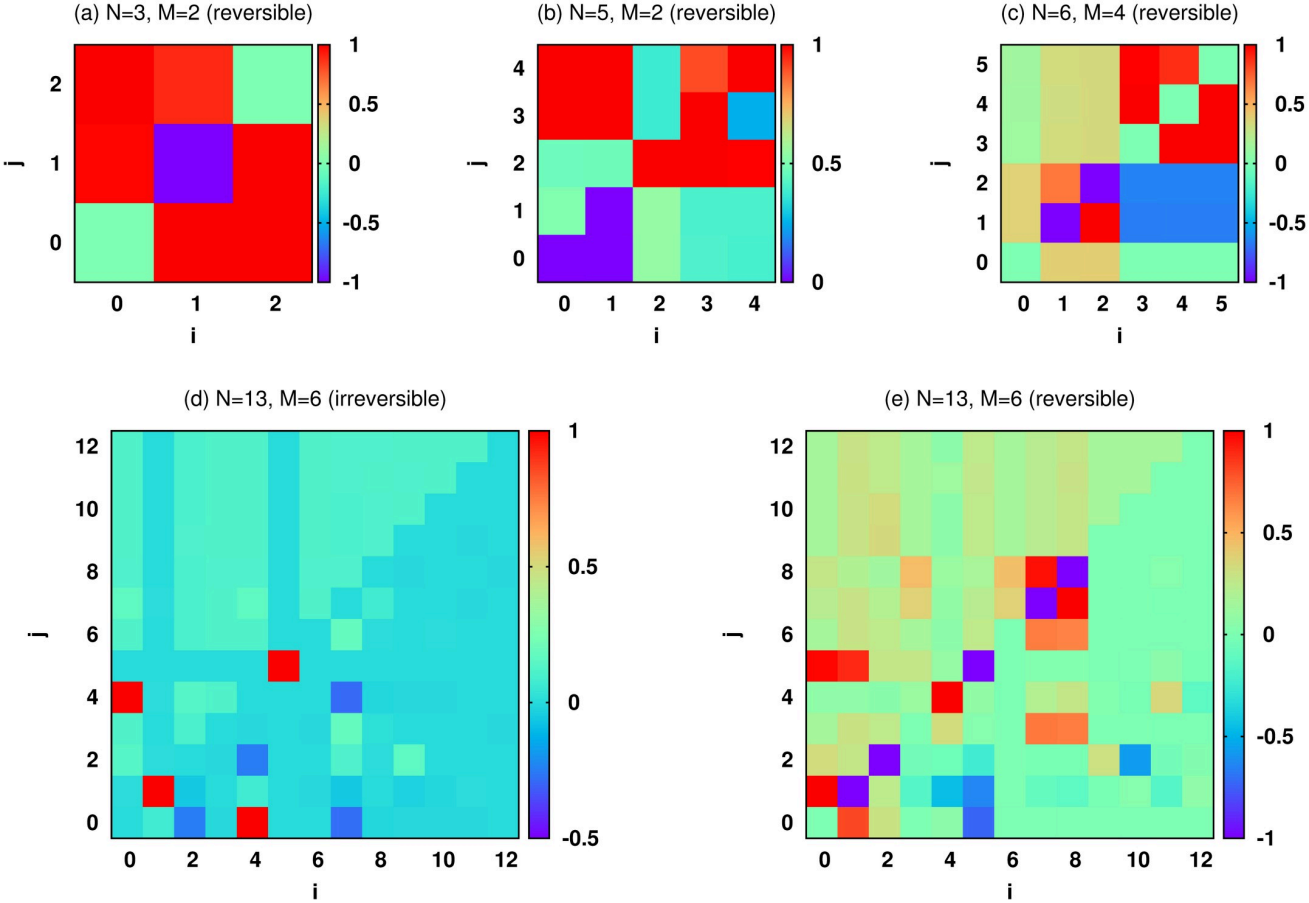
Statistical characterization of the optimal (healthy) reaction networks. The matrix displays mutual informations MI(*i*, *j*) (the upper triangle of the matrix), correlations coefficients *C*(*i*, *j*) (the lower triangle of the matrix), and concentration values *x*_*i*_ (the diagonal elements) for the reaction networks of Fig 2(a), 2(d), 2(f) and 2(j): (a) the single chain $\left(S=X_{0},R\left(X_{0}\right)=X_{2}\right)$, (b) the two-chain network with two common reactions $\left(S=\left\{X_{0},X_{1}\right\},R\left(X_{0}\right)=X_{3},R\left(X_{1}\right)=X_{4}\right)$, (c) the two-chain network with a connecting link $\left(S=\left\{X_{0},X_{3}\right\},R\left(X_{0}\right)=X_{2},R\left(X_{3}\right)=X_{5}\right)$, (d) and (e) the three-chain network with reversible and irreversible reactions $\left(S=\left\{X_{0},X_{3},X_{6}\right\},R\left(X_{0}\right)=X_{2},R\left(X_{3}\right)=X_{5},R\left(X_{6}\right)=X_{8}\right)$. The MI(*i*, *j*) and *C*(*i*, *j*) are scaled to have maximum value one.

Fig 4 shows the optimal features of some selected reaction networks (from Fig 2) obtained in this way. The figure displays the coarse-grained activities *x*_*i*_, the mutual information *MI*(*i*, *j*), and the correlation coefficients *C*(*i*, *j*). Note that the coefficients *C*(*i*, *j*) display only linear correlations between *X*_*i*_ and *X*_*j*_. Nevertheless, it is easier to say that the two variables are positively or negatively correlated by looking at the sign of *C*(*i*, *j*) than the value of mutual information. We observe in the figure that negative correlations appear in networks of interacting pathways due to the presence of feedback loops; a single pathway like the one on panel (a), displays merely positive correlations as expected. Moreover, due to the same interactions in the reversible network of panel (e), we see that by maximizing the above objective function we also obtain considerable cross mutual informations, e.g., for MI(*X*_0_, *X*_5_) and MI(*X*_3_, *X*_8_).

The optimized reaction network gives the initial condition for the time evolution of the model with defects **D** as follows. At each step *t*, we change the *κ*_*r*_ to *κ*_*r*_ ± *δκ* with probability *α*(*t*) = min(1, *t*/*τ*_*α*_) for the reactions that are affected by **D**. Here *δκ* = 0.05, *τ*_*α*_ = 100, 400, and still we have the constraint that *κ*_*r*_ ∈ (0, 2). The other parameter decreases with time step *t* as *β*(*t*) = *max*(0, *β*_0_(1 − *t*/*τ*_*β*_)), where *β*_0_ = 100 and *τ*_*β*_ = 100, 400. The mutual informations after each update of the reaction rates are estimated by the stochastic evolution of the reaction network for Δ*t*_*eq*_ = 200 iterations. Then, we run the algorithm for another Δ*t*_*av*_ = 200 iterations to extract the necessary statistical information. Note that all parameters here are dimensionless and should be scaled to be related to the real quantities. The parameters like the reaction rates and the time scales are chosen to display the typical behaviour of the system in different regimes. To be specific, in the following we focus on the temporal evolution of diseases in the larger reaction network of Fig 2(j).

**Fig 5:**
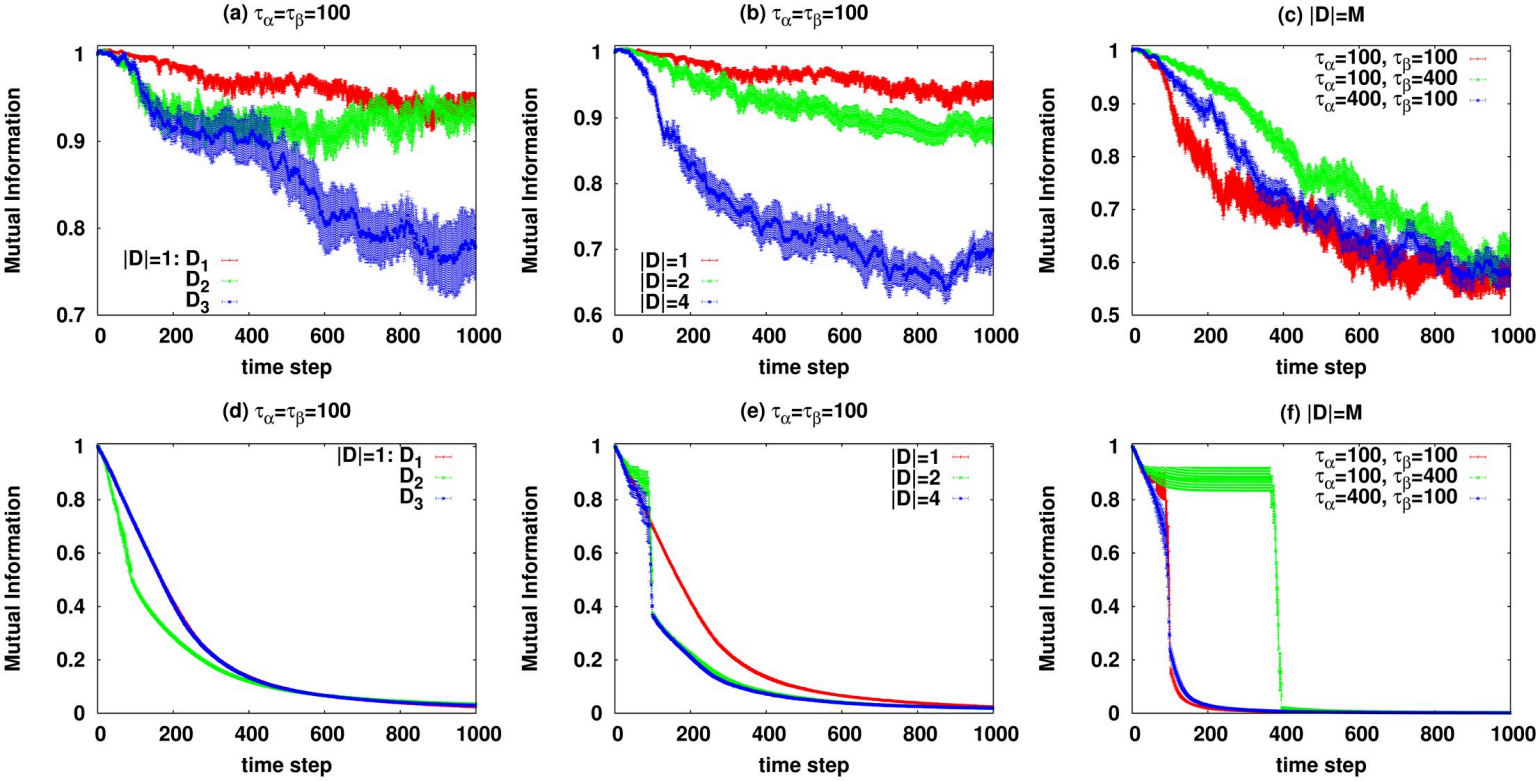
The average objective function (scaled to have maximum one) in presence of various defects. The data are for the reaction network of Fig 2(j) with *N* = 13 species and *M* = 6 reactions. Top panels show the results with reversible reactions for: (a) evolution with one defect (|**D**| = 1) for *τ*_*α*_ = *τ*_*β*_ = 100, (b) evolution with one, two, and four defects (|**D**| = 1, 2, 4) for *τ*_*α*_ = *τ*_*β*_ = 100, (c) evolution with all defects (|**D**| = *M*) for different *τ*_*α*_ = 100, 400 and *τ*_*β*_ = 100, 400. Bottom panels (d),(e),(f) show the results with irreversible reactions.

**Fig 6:**
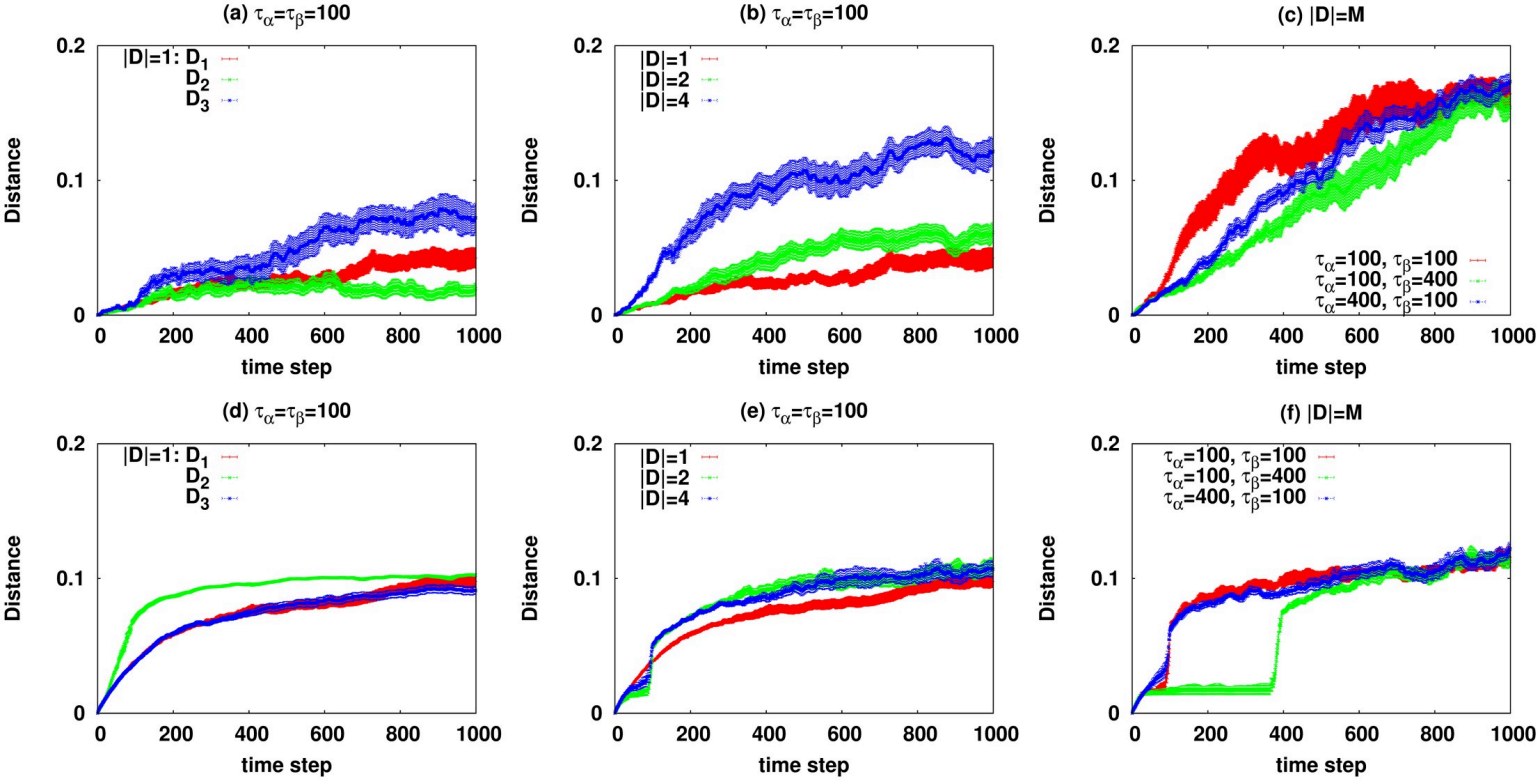
The average distance *d*(*t*) = ∑_*i*_|*c*_*i*_(*t*) − *c*_*i*_(0)|/*N* of the concentration values *c*_*i*_(*t*) = 〈*X*_*i*_(*t*)〉/*X*_*max*_ at different time steps. The data are for the reaction network of Fig 2(j) with *N* = 13 species and *M* = 6 reactions. Top panels show the results with reversible reactions for: (a) evolution with one defect (|**D**| = 1) for *τ*_*α*_ = *τ*_*β*_ = 100, (b) evolution with one, two, and four defects (|**D**| = 1, 2, 4) for *τ*_*α*_ = *τ*_*β*_ = 100, (c) evolution with all defects (|**D**| = *M*) for different *τ*_*α*_ = 100, 400 and *τ*_*β*_ = 100, 400. Bottom panels (d),(e),(f) show the results with irreversible reactions.

Figs 5 and 6 display the average objective function (mutual information) and distance from the healthy state we observe for some defect patterns **D**. Here the average is taken over at least 100 realizations of the stochastic disease dynamics. The numerical results for the behaviour of single dynamical realizations are given in S1 Text. As the figures show, besides the number of present defects which is displayed in panels (b),(e), it is the relative difference of the two time scales *τ*_*α*_ and *τ*_*β*_ that determines the qualitative behaviour of the system. For instance, in panels (c),(f) of the figures we observe that the transition from the healthy state is usually sharper when the rate of mutations 1/*τ*_*α*_ is smaller than the rate of accepting the mutations; it makes sense as in this case the probability of accepting the changes is already considerable when the chance of happening the mutations becomes large. On the other hand, a two-stage behaviour is observed in panels (c),(f) when the rate of mutations is significantly larger than 1/*τ*_*β*_. Here, there is a period where the mutations happen frequently but the rate of accepting the mutations is small and they are mostly rejected. Fig 6 shows the distance of the system state at time step *t* from the initial state $d\left(t\right)=\sum_{i=1}^{N}\left|c_{i}\left(t\right)-c_{i}\left(0\right)\right|/N$, where *c*_*i*_(*t*) = 〈*X*_*i*_(*t*)〉/*X*_*max*_. The figure shows the times that the system spends around a microscopic state and how the distance changes in presence of various defects.

**Fig 7:**
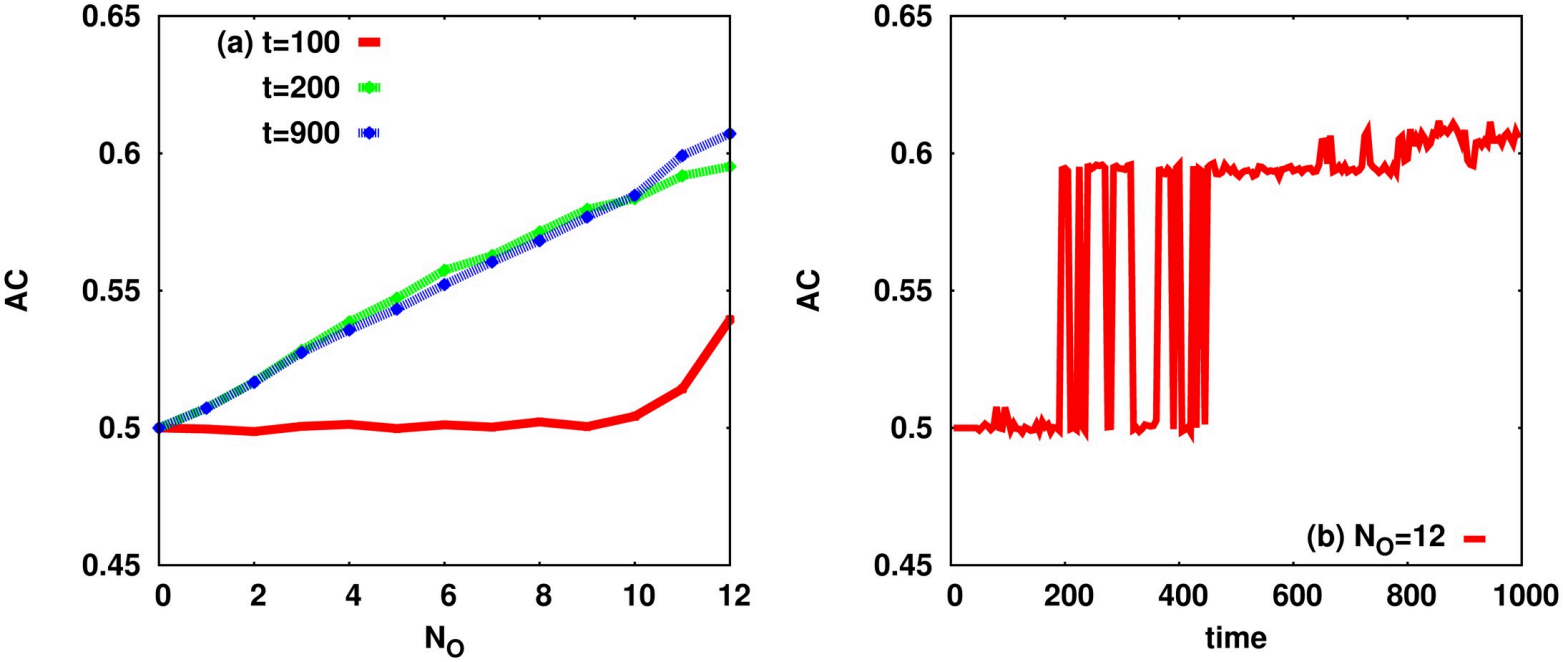
Accuracy of predictions with the probabilistic diagnostic model of Ref [14]. The accuracy (true positive + true negative)/(total positive + total negative) at different time steps *t* with *τ*_*α*_ = *τ*_*β*_ = 100: (a) Using the D1S1 model in the presence of one defect (|**D**| = 1) and (b) Time dependence of the accuracy in presence of one defect (|**D**| = 1) for *N*_*O*_ = 12.

Now, we consider the temporal behaviour of the diagnosis accuracy in the above reaction network with reversible reactions. The disease evolution algorithm is run for various elementary defects to obtain the binary sign variables *S*_*i*_(*t*) = ±1 from the molecular numbers *X*_*i*_(*t*). Let us assume that the value of *N*_*O*_ signs are observed at time step *t*. The aim then is to find out the elementary defect that resulted in the observed values. Fig 7 shows the average accuracy of the predictions made by the D1S1 model of Ref [14], assuming that |**D**| = 1 and *τ*_*α*_ = *τ*_*β*_ = 100. To construct the probabilistic diagnostic model we need the conditional probabilities Pr_*t*_(*S*_*i*_|nodefect), and Pr_*t*_(*S*_*i*_|*D*_*r*_) at time step *t*. This information is obtained from numerical simulation of the dynamics of the reaction network in the healthy state (no defect), and in presence of only one defect (*D*_*r*_), respectively. The reported accuracy is averaged over different elementary defects and dynamical realizations.

The accuracy of diagnosis is expected to improve with the evolution time of the defects; obviously, there is more statistical information about the defects in the data that are extracted at larger times and this enhances the ability to distinguish between different defects. We observe in Fig 7(a) that this improvement (with respect to random predictions) in the accuracy is very sharp; it is nearly zero for times less than 200 and then it takes a nearly constant value (for given *N*_*O*_) at larger times. The figure (panel (b)) also shows how the accuracy (for a given *N*_*O*_ = 12) changes with time when the number of present defects |**D**| = 1. Here it is clearer to see the discontinuous behaviour of the average accuracy; in particular, there is a transition time interval (200, 400) where the system displays two macroscopic states with different values for the diagnostic accuracy. When the accuracy exhibits such a behaviour, the optimal intervention time for diagnosis, considering the decreasing objective function, is just after the transition.

Note that here we are using only the statistical information that are obtained at a given time step *t*. A more accurate diagnosis should take into account the whole history of the sign variables, which is the subject of our future studies.

## Discussion

In summary, we presented a stochastic model using the reverse annealing algorithm for the simulation of disease evolution in time. The dynamical model depends on two parameters, which control the rate of introducing mutations (defects) and the rate of accepting a decrease in the objective function. The relative strength of these parameters determines the overall behaviour of the system. For instance, the transition to the disease state is sharper when the rate of generating a mutation is lower than the rate of accepting the mutation. Moreover, we used a probabilistic diagnostic model to estimate the accuracy of diagnosis as the system performance degrades in time in the presence of some defects. The results show how much the diagnostic accuracy improves by the elapsed time of a disorder. This allows us to quantify the tradeoff between the accuracy of diagnosis and the degree of disease progression.

A microscopic model of disease evolution could be useful for a diagnostic problem which is to consider the history (dynamics) of the sign variables **S**(*t*_*i*_ → *t*_*f*_) = {**S**(*t*_*i*_), …, **S**(*t*_*f*_)}. For instance, given the time dependence of the molecular concentrations *X*_*i*_(*t*) in a chemical reaction network, then a relevant problem is to reconstruct the time evolution of the model parameters *α*_*r*_(*t*), *β*(*t*). Obviously, a diagnosis that relies on the likelihoods of defects for a given history **S**(*t*_*i*_ → *t*_*f*_), would be more accurate than a diagnosis that is solely based on the current sign values.

The model presented in this study is of course a toy model, which does not recapitulate fully the biological complexity of disease characterization and development. However, we believe that it could be a good starting model to capture some of the essential ingredients of disease progression and the clinical diagnostic problem. The main objectives behind this work are:

- to provide a microscopic model that generates synthetic data which are needed for construction of deeper probabilistic models of signs and diseases.
- to present a dynamical model of disease evolution which (I) can be used to model explicitly disease-disease interactions and (II) can be used directly in a diagnostic problem that is based on the dynamics or history of the observed signs. That is to infer the effective parameters *α*_*r*_ and *β* to uncover the underlying defects behind the dynamics.

As a first application, we used the proposed simple diagnostic model to provide insights into the optimal intervention time (considering the accuracy of diagnosis and the level of disease development). The second application can involve, for example, a well reconstructed part of the cell reaction network with a history of observed molecular concentrations, to localize the position of diverging reactions in the network from the inferred parameters *α*_*r*_. On the other hand, a global measure of the network deviation from the healthy state is provided by the parameter *β* which somehow quantifies the overall level of destructive noises in the reaction network.

It would be interesting to apply the methodology of this study to the Hopfield model of associative neural networks [35]. In addition to connections with the study of mental disorders, the Hopefield model is also related to simple models of the immune system [36, 37]. Finally, the study can also be done for a personal reaction network which has been reconstructed from a single-person biomedical data [38–40]. This, in turn, would allow for a more precise and personalized diagnosis.

## Methods

The results of numerical simulations are obtained for the reaction networks of Fig 2. The reaction networks are chosen in a way that mimic the biological structures. The number of molecules for each species is restricted to 0 ≤ *X*_*i*_(*t*) < *X*_*max*_ = 1000. The value of *X*_*max*_ is chosen to be of the order of the molecular numbers in real biological systems. The initial number of molecules is chosen randomly and uniformly in (0, *X*_*max*_). For the externally driven species, which are indicated by dashed arrows in the figure, we assume that the number of molecules at any time obeys a uniform probability distribution. It means that the number of these species is not determined by the system dynamics but these species still affect the reactions in which they play the role of reactants. To compute the probabilities and mutual informations we do the numerical simulation in parallel for a population of $N_{pop}=10^{5}$ independent and identical reaction networks.

We use a zero-temperature Monte Carlo algorithm to find a local maximum of the objective function. We start from random initial values for the *κ*_*r*_. At each step, new reaction rates are suggested around the current values. Then, the mutual informations are estimated from the stochastic dynamics of the reaction network for $\Delta t_{eq}^{0}=4000$ iterations to reach a stationary state. We run the algorithm for another $\Delta t_{av}^{0}=1000$ iterations to extract the statistical information needed for computation of the objective function; at each iteration, we allow for *M* reactions to happen according to the dynamical rules of the stochastic evolution (Gillespie) algorithm. The suggested changes in the reaction rates are accepted only if the objective function increases.

## Supporting information

S1 Text Supporting information are provided in file S1_Text. (PDF)
