# Supplementary material for: Disease evolution in reaction networks: Implications for a diagnostic problem
Source: PLoS Comput Biol. 2020 Jun 4;16(6):e1007889. doi: 10.1371/journal.pcbi.1007889 (PMC7272006; doi:10.1371/journal.pcbi.1007889)
Supplement: S1 Text — (PDF) [file pcbi.1007889.s001.pdf]

Disease evolution in reaction networks: Implications for a diagnostic problem

Abolfazl Ramezanpour[1,2], Alireza Mashaghi[1*],

1 Medical Systems Biophysics and Bioengineering, Leiden Academic Centre for Drug Research, Faculty of Science, Leiden University, Leiden, The Netherlands
2 Physics Department, College of Sciences, Shiraz University, Shiraz, Iran

* a.mashaghi.tabari@lacdr.leidenuniv.nl

Supporting information

We take the reaction network of three interacting pathways with $N = 13$ species and $M = 6$ reactions shown in Fig A(j). In the main manuscript, we reported the objective function and the accuracy of diagnosis averaged over different dynamical realizations of disease evolution. The following figures show the results obtained by a single realization of the stochastic dynamics. The models, computational methods, and parameters are the same as in the main manuscript.

Figure B displays the objective function (mutual information) we obtain for different defect patterns $\mathbf{D}$. As the figure shows, besides the number of present defects, it is the relative difference of the two time scales τ_α and τ_β that determines the qualitative behaviour of the system. Figure C shows the distance of two system states at two time steps $d(t_1, t_2) = \sum_{i=1}^{N} |c_i(t_2) - c_i(t_1)|/N$ with $c_i(t) = \langle X_i(t) \rangle / X_{max}$. The size of diagonal blocks give the time periods that the system spends around a microscopic state. This is to see how the macroscopic state of the system changes in presence of the defects. Figure D shows the accuracy of the predictions made by the diagnostic model D1S1 of Ref [1], for a given number of observed signs N_O. We see how the accuracy of diagnosis improves (as expected) with the evolution time of the defects. The figure also shows how the normalized objective function and accuracy (for given $N_O = 12$) change with time when the number of present defects $|\mathbf{D}| = 1$. In contrast to the average accuracy that is reported in the main text, here the accuracy is changing more smoothly with time, probably due to larger single realization fluctuations.

References

1. Ramezanpour A, Mashaghi A. Toward First Principle Medical Diagnostics: On the Importance of Disease-Disease and Sign-Sign Interactions. Frontiers in Physics. 2017 Jul 25;5:32.

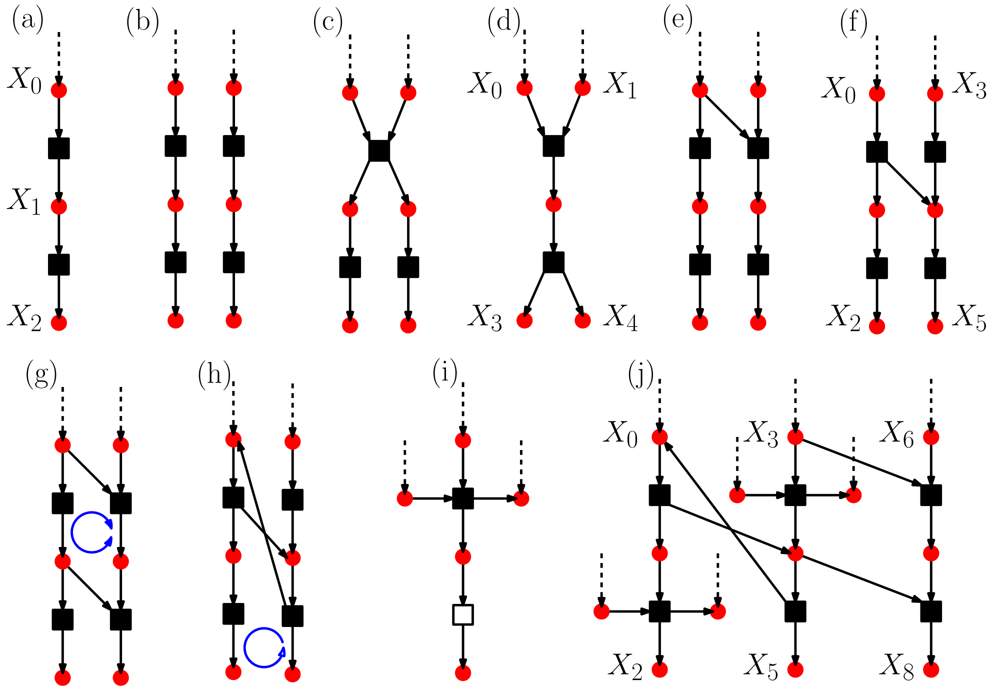

Fig A. Illustration of the reaction networks. The solid circles and squares display the species and the reactions, respectively. A reaction could be reversible (full square) or irreversible (empty square). A dashed arrow shows that the species concentration is driven externally. (a) a single pathway, (b) two non-interacting pathways, (c)-(d) two pathways interacting through common reactions and species, (e)-(f) two pathways interacting through a link, (g)-(h) two pathways interacting through a coherent (h) or incoherent (g) cycle, (i) a pathway with an irreversible reaction which also interacts with two externally driven species, (j) a larger reaction network of interacting pathways with $M = 6$ reactions and $N = 13$ species.

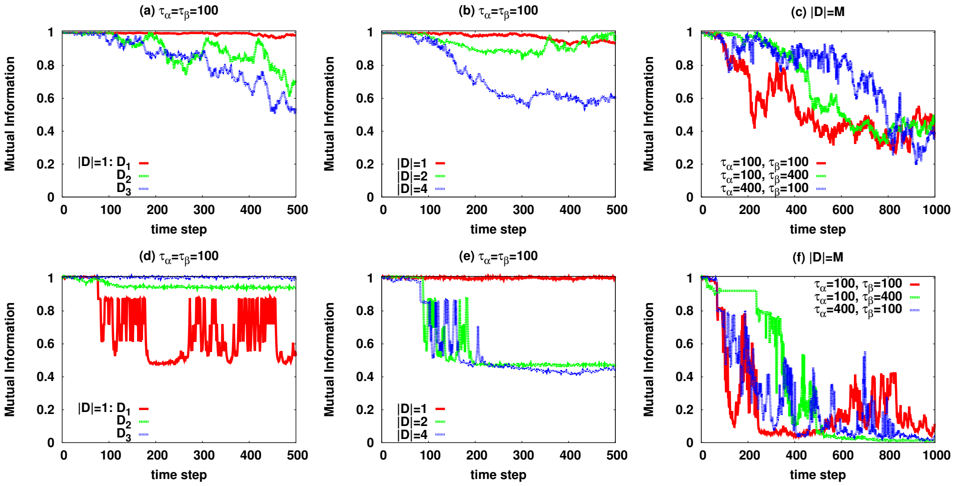

Fig B. Typical behaviours of the objective function (scaled to have maximum one) in the presence of defects. The data are for the reaction network of Fig A(j) with $N = 13$ species and $M = 6$ reactions. Top: The results with reversible reactions for (a) evolution with one defect ($|\mathbf{D}| = 1$) for $\tau_\alpha = \tau_\beta = 100$, (b) evolution with one, two, and four defects ($|\mathbf{D}| = 1, 2, 4$) for $\tau_\alpha = \tau_\beta = 100$, (c) evolution with all defects ($|\mathbf{D}| = M$) for different $\tau_\alpha = 100, 400$ and $\tau_\beta = 100, 400$. Bottom: The panels (d),(e),(f) show the results with irreversible reactions.

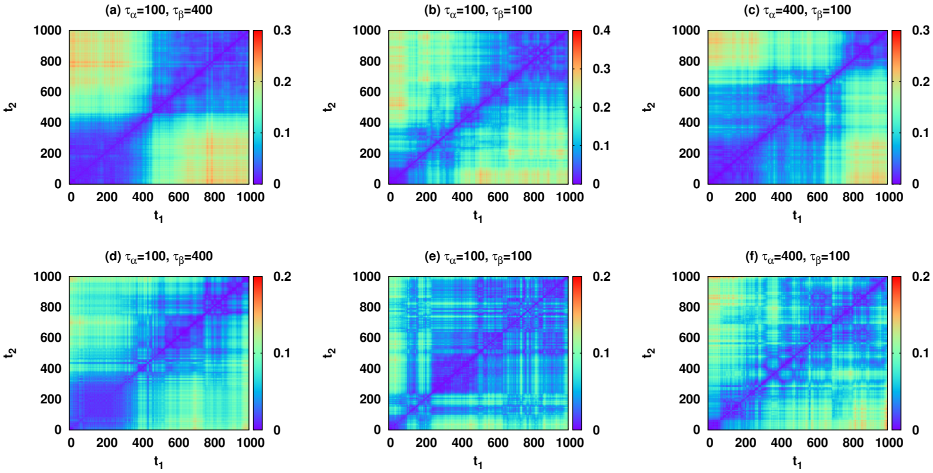

Fig C. Matrix plot of differences in concentrations at different time steps. The matrix elements show distances $d(t_1, t_2) = \sum_i |c_i(t_2) - c_i(t_1)|/N$ of the concentration values $c_i(t) = \langle X_i(t) \rangle / X_{max}$ at different time steps. The data are for the reaction network of Fig A(j) with $N = 13$ species and $M = 6$ reactions. Top: The results with reversible reactions when all the defects are present ($|\mathbf{D}| = M$) with (a) $\tau_\alpha = 100, \tau_\beta = 400$, (b) $\tau_\alpha = 100, \tau_\beta = 100$, and (c) $\tau_\alpha = 400, \tau_\beta = 100$. Bottom: The panels (d),(e),(f) show the results with irreversible reactions.

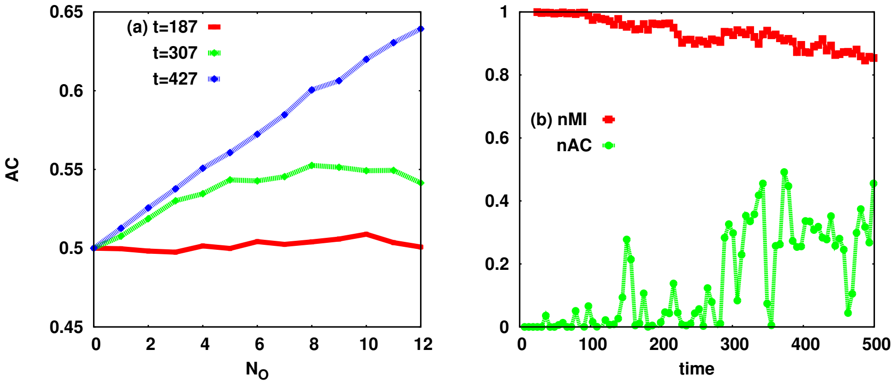

Fig D. Accuracy of predictions with the probabilistic diagnostic model (D1S1) of Ref [1]. The accuracy (true positive + true negative)/(total positive + total negative) at different time steps t with $\tau_\alpha = \tau_\beta = 100$: (a) Using the D1S1 model in the presence of one defect ($|\mathbf{D}| = 1$) and (b) Time dependence of the normalized objective function and accuracy in the presence of one defect ($|\mathbf{D}| = 1$) for $N_O = 12$.
